# Oral dextran sulfate sodium administration induces peripheral spondyloarthritis features in SKG mice accompanied by intestinal bacterial translocation and systemic Th1 and Th17 cell activation

**DOI:** 10.1186/s13075-022-02844-4

**Published:** 2022-07-25

**Authors:** Yuya Tabuchi, Masao Katsushima, Yuri Nishida, Mirei Shirakashi, Hideaki Tsuji, Hideo Onizawa, Koji Kitagori, Shuji Akizuki, Ran Nakashima, Kosaku Murakami, Koichi Murata, Hajime Yoshifuji, Masao Tanaka, Akio Morinobu, Motomu Hashimoto

**Affiliations:** 1 Department of Rheumatology and Clinical Immunology, Graduate School of Medicine, Kyoto University, Konoe-cho, Sakyo-ku, Kyoto, Japan; 2 Department of Advanced Medicine for Rheumatic Diseases, Graduate School of Medicine, Kyoto University, Konoe-cho, Sakyo-ku, Kyoto, Japan; 3 Center for Cancer Immunotherapy and Immunobiology, Graduate School of Medicine, Kyoto University, Konoe-cho, Sakyo-ku, Kyoto, Japan; 4 Department of Clinical Immunology, Graduate School of Medicine, Osaka Metropolitan University, 1-4-3, Asahi-machi, Abeno-ku, Osaka, Japan

## Abstract

**Background:**

Spondyloarthritis (SpA) is an autoimmune and autoinflammatory musculoskeletal disease characterised by systemic enthesitis. Recent research has focused on subclinical inflammatory bowel disease (IBD) in SpA pathogenesis. SKG mice, harbouring the Zap70 W163C mutation, increase autoreactive Th17 cells intrinsically, and in a conventional environment, they exhibit spontaneous arthritis with fungal factors. Under SPF conditions, they show SpA features, including enteritis, after peritoneal injection of β-1,3-glucan. This study aimed to clarify whether oral dextran sulfate sodium (DSS) administration, utilised in IBD model mice, can provoke SpA features in SKG mice under SPF conditions, focusing on the relationship between gut microorganisms and SpA pathogenesis.

**Methods:**

BALB/c and SKG mice were administered oral DSS, and their body weights, arthritis, and enthesitis scores were recorded. In another cohort, antibiotics (meropenem and vancomycin) or an anti-fungal agent (amphotericin B) was administered orally before DSS administration. The splenic Th1 and Th17 cell populations were examined before and after DSS administration using flow cytometry. Furthermore, the amount of circulating bacterial DNA in whole blood was measured by absolute quantitative polymerase chain reaction (qPCR), and the number and characteristics of bacterial species corresponding to these circulating DNA were analysed by next-generation sequencing (NGS).

**Results:**

Ankle enthesitis as a peripheral SpA feature was elicited in half of DSS-administered SKG mice, and none of the BALB/c mice. Pre-administration of antibiotics suppressed enthesitis, whilst an anti-fungal agent could not. Th1 and Th17 cell levels in the spleen increased after DSS administration, and this was suppressed by pre-administration of antibiotics. SKG mice have a larger amount of bacterial DNA in whole blood than BALB/c mice before and 1 day after the initiation of DSS administration. The number of bacterial species in whole blood increased after DSS administration in BALB/c and SKG mice. Some genera and species significantly specific to the DSS-treated SKG mouse group were also detected.

**Conclusion:**

Oral DSS administration alone elicited peripheral enthesitis in SKG mice with bacterial translocation accompanied by increased splenic Th1 and Th17 cell levels. Pre-administration of antibiotics ameliorated these DSS-induced SpA features. These findings suggest that intestinal bacterial leakage plays a pivotal role in SpA pathogenesis.

## Introduction

Spondyloarthritis (SpA) is an autoimmune and autoinflammatory musculoskeletal (MSK) disease characterised by systemic enthesitis [1], comprising ankylosing spondylitis (AS), psoriatic arthritis (PsA), inflammatory bowel disease (IBD)-associated arthritis, and reactive arthritis (ReA) [2]. Genetic backgrounds, including HLA-B27 positivity, mechanical stress, and dysbiosis, have been investigated in the pathogenesis of SpA [3]. In addition, subclinical IBD has recently attracted attention. According to some reports, approximately half of patients with AS have subclinical microscopic IBD [4]. Another report has shown an increase in faecal calprotectin levels in patients with AS [5]. An increasing number of researchers are studying the relationship between MSK manifestations and gut inflammation in the pathogenesis of SpA [6]. However, there are many unrevealed disease mechanisms.

Animal models are essential for clarifying the complicated pathogenesis of SpA. SKG mice, identified by Sakaguchi et al., possess the Zap70 W163C mutation [7]. In a conventional environment, they are known for their intrinsically increased autoreactive Th17 cells and spontaneous IL-17A-dependent autoimmune inflammatory arthritis [8]. Reports show that both spontaneous arthritis in SKG mice under conventional conditions and β-glucan-induced arthritis under specific pathogen-free (SPF) conditions are related to the dectin-1 pathway. Furthermore, intraperitoneal injection of an anti-fungal agent, amphotericin B, prevents the onset of arthritis completely in a conventional environment and partially under SPF conditions [9]. It has also been shown that intraperitoneal injection of curdlan, β-1,3-D-glucan, causes MSK SpA features and IBD-like enteritis in SKG mice under SPF conditions [10]. The same group has shown that the Zap70 mutation in SKG mice disrupts the homeostatic relationship between gut microbiota and the host, and that SKG mice have intrinsic gut dysbiosis due to their genetic background [11]. They also demonstrated that intestinal IL-23 plays a pivotal role in the pathogenesis of β-glucan-induced SpA pathogenesis in SKG mice [12]. However, little is known about whether oral administration of dextran sulfate sodium (DSS), which is commonly used in studies regarding IBDs, has the potential to cause SpA features, including peripheral enthesitis in SKG mice, and if so, what the mechanisms are like.

This study investigated whether oral DSS administration under SPF conditions solely provokes enthesitis as a SpA feature in SKG mice, focusing on the relationship between bacterial and/or fungal translocation and SpA pathogenesis.

## Methods

### Mice, induction of disease, and reagents

BALB/c mice were purchased from Claire Japan, Inc. (Tokyo, Japan). SKG mice were obtained from Prof. Sakaguchi (Osaka University, Osaka, Japan). Both mice were bred under SPF conditions in the main facility of the Institute of Laboratory Animals, Kyoto University. All experiments were approved by the Kyoto University Animal Ethics Committee. In all experiments, only female mice 8 to12 weeks of age were used, since previous reports have shown that female SKG mice have higher clinical scores than male [10, 13]. In the DSS administration cohorts, 1% DSS (MP Biomedicals, Santa Ana, CA, USA, molecular weight 35,000–50,000 kDa) in drinking water was administered for the first two consecutive weeks. In the set of cohorts with antibiotic or anti-fungal agents, 6% dimethyl sulfoxide (DMSO) (Nacalai Tesque Inc., Tokyo, Japan) as a control group, 6% DMSO and meropenem trihydrate (MEPM) (1 g/L) (FUJIFILM Wako Chemicals Inc., Tokyo, Japan) and vancomycin hydrochloride (VCM) (0.5 g/L) (Nacalai Tesque Inc.), and 6% DMSO and amphotericin B (AMPH-B) (0.3 g/L) (Nacalai Tesque Inc.) were administered for 1 week in drinking water, from the time point 2 weeks before the initiation of DSS administration. Ten mice were used per group to confirm disease phenotypes, and 5 mice were used per group in other cohorts for the FCM, qPCR, and NGS analyses.

### Histologic analysis

The ankle enthesis and caudal vertebral joints from BALB/c and SKG mice with or without DSS treatment were fixed in formalin and embedded in paraffin at the experimental endpoints. A mouse with swollen ankles was selected for the representative photograph of DSS-treated SKG mice. Four-micrometre sections were cut and stained with haematoxylin and eosin (H&E). Photographs were taken with an Olympus CKX31 microscope and an Olympus STYLUS 1s camera.

### Scoring of clinical signs

**Fig. 1:**
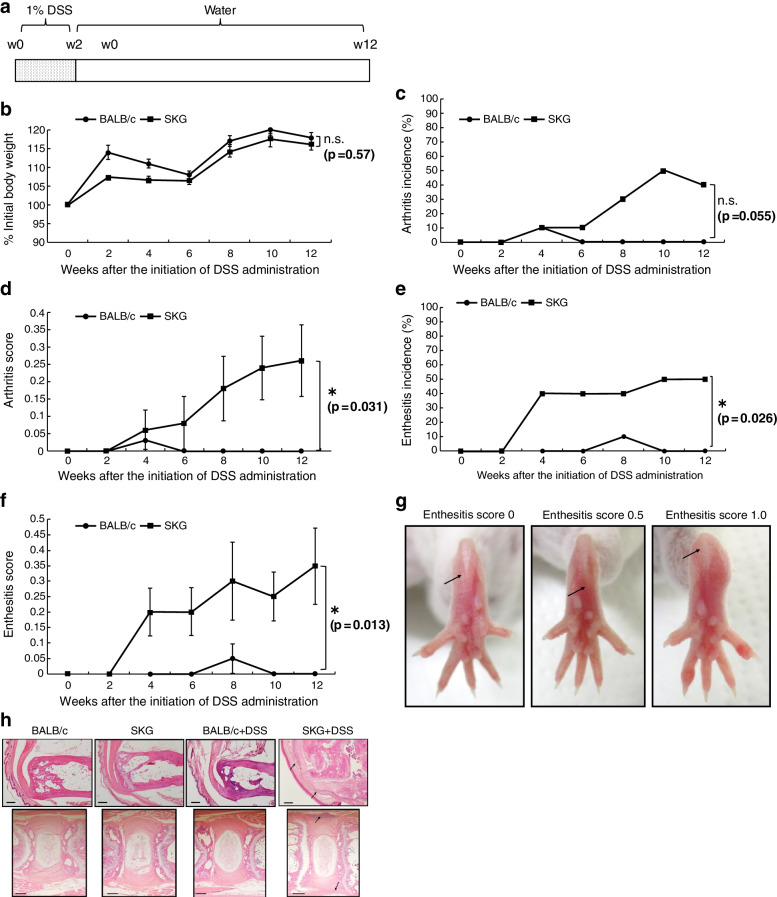
Oral dextran sodium sulfate (DSS) administration elicits SpA features in SKG mice. **a** Experimental scheme of oral DSS administration during the experiment. Mice were given 1% DSS in their drinking water for 2 weeks, followed by regular water. Ten mice were used in each group. Weeks are indicated by w. **b** The body weight change in BALB/c and SKG mice over the experiment. **c**–**f** Arthritis incidence rates and scores and enthesitis incidence rates and scores were recorded every 2 weeks. Values are expressed as mean ± standard error of the mean (SEM). **g** Representative photographs of hind paws for the enthesitis evaluation method. **h** Representative haematoxylin and eosin-stained sections of the peripheral enthesis (Achilles tendon) and the axial enthesis (caudal vertebrae). Only DSS-treated SKG mice showed marked cell infiltration around the Achilles tendon and plantar aponeurosis (arrows), and slight cell infiltration around but not inside the annulus fibrosus of the intervertebral disk (arrows). Scale bar = 200μm. Statistical analyses were performed by using the Mann-Whitney *U* test (*P <0.05)

Clinical features of the mice were scored every other week by the same observer. A conventional scoring system by Sakaguchi et al. was used to evaluate arthritis [7]. The ankle was scored on each side to evaluate enthesitis, based on our original criteria: 0.5 = deviation of the plantar aponeurosis (the midline of the plantar aponeurosis indicating a toe other than the third one) and 1.0 = uplift of the plantar aponeurosis over the angle of the ankle (Fig. 1g). The scores of both ankles were summed, and the maximum possible enthesitis score was 2.0.

### Flow cytometry

Single-cell suspensions were prepared by triturating the spleens between the ends of sterile frosted slides and filtration through nylon mesh. After erythrocyte lysis, splenocytes were suspended in RPMI 1640 tissue medium supplemented with 10% heat-inactivated foetal bovine serum, 50 U/mL penicillin, and 50 μg/mL streptomycin, and cultured for 3 h at 37 °C with Cell Stimulation Cocktail (eBioscience, CA, USA). After FcγR blockade with anti-CD16/32 antibodies (BioLegend, CA, USA), extracellular antigens were stained for 20 min at 4 °C in RPMI 1640 medium. Cells were fixed and permeabilised using Foxp3/Transcription Factor Buffer Set (eBioscience) and stained for intracellular cytokines. Samples were acquired using a BD FACSCalibur flow cytometer operated by CellQuest (BD Biosciences, CA, USA), and the data were analysed using FlowJo software (BD Biosciences). Cell populations were identified by staining with anti-CD3-FITC (17A2), anti-CD4-PerCp/Cy5.5 (GK1.5), anti-IL-17A-PE (TC11-18H10), and anti-IFN-γ–Alexa Flour 647 (XMG1.2) (BioLegend).

### DNA extraction and absolute quantitative polymerase chain reaction

Whole blood (150 μL) was collected in DNA-free tubes from each mouse using a Goldenrod Animal Lancet (Braintree Scientific Inc., MA, USA), quickly preserved at 4 °C, and then frozen at −30 °C until DNA extraction. NucleoSpin Blood QuickPure (MACHEREY-NAGEL GmbH & Co. KG, Dueren, Germany) was used for the DNA extraction. Absolute qPCR was performed, using a Femto Bacterial DNA Quantification Kit (Zymoresearch, Inc., CA, USA) and an AB 7500 machine (Applied Biosystems, Inc., MA, USA) according to the manufacturers’ indication. Samples with a Ct value more than 35 cycles or undetectable were counted as 0 pg/mL.

### NGS-based 16S gene analysis

Extracted DNA from whole blood was amplified on the whole 16S coding region (V1-9) by PCR, using the Bacterial 16S rDNA PCR Kit (Takara Bio Inc., Shiga, Japan) and MiniAmp Plus Thermal Cycler (Applied Biosystems). The amplicons were cleaned using NucleoSpin Gel and PCR Clean-up (MACHEREY-NAGEL GmbH & Co. KG). The purified amplicons were amplified again on the 16S V3-V4 region, using Phusion High-Fidelity PCR Master Mix (New England Biolabs, Inc., MA, USA) with specific primers and barcodes. The same volume of loading buffer (containing SYBR green) was mixed with PCR products, and electrophoresis on a 2% agarose gel was performed. Samples with a bright main strip between 400 and 450bp were chosen for further experiments. PCR products were purified using the Qiagen Gel Extraction Kit (Qiagen GmbH, Hilden, Germany). The libraries were generated using NEBNext UltraTM DNA Library Prep Kit (New England Biolabs) and sequenced on an Illumina NovaSeq6000 (Illumina, Inc., CA, USA). Paired-end reads were assigned to samples based on their unique barcodes and truncated by cutting off the barcode and primer sequences. Paired-end reads were merged using FLASH (V1.2.7). Quality filtering on the raw tags was performed under specific conditions to obtain high-quality clean tags according to QIIME (V1.7.0). The tags were compared with the reference database using the UCHIME algorithm to detect chimaera sequences, and chimaera sequences were removed. Analysis of similarities (Anosim) was performed using QIIME software (Version 1.7.0). LEfSe analysis was conducted using the LEfSe software.

### Statistical analysis

All statistical analyses were performed using JMP Pro 15 or EZR (Version 1.40), except for the NGS analyses. Statistical tests performed for each dataset and *p* values and significance levels are indicated in the figures.

## Results

### Oral DSS administration triggers a peripheral SpA phenotype in SKG mice

To investigate whether oral DSS administration solely provokes SpA features in SKG mice under SPF conditions, female BALB/c and SKG mice aged 8–12 weeks (*n*=10 per group) were administered 1% DSS in drinking water for 2 weeks under SPF conditions (Fig. 1a). Body weights, arthritis, and enthesitis scores were recorded every 2 weeks over 12 weeks. At week 12, there was no significant difference in the rate of body weight change between BALB/c and SKG mice (Fig. 1b). The incidence rates of arthritis and enthesitis at week 12 were 40% and 50% in the SKG mouse group, respectively (Fig. 1c, e). Arthritis and enthesitis composite scores in the SKG mice increased chronologically, whilst none of those of the BALB/c mice did (Fig. 1d, f). In the SKG mice, deviation and/or uplift of the plantar aponeurosis over the angle of the ankle was confirmed (Fig. 1g). H&E staining revealed outstanding cell infiltration at the Achilles tendon and plantar aponeurosis insertions in the DSS-treated SKG mice. Slight cell infiltration adjacent to the vertebral disk was also confirmed in the DSS-treated SKG mouse group (Fig. 1h).

### Oral DSS activates both Th1 and Th17 immunity in DSS-treated SKG mice

**Fig. 2:**
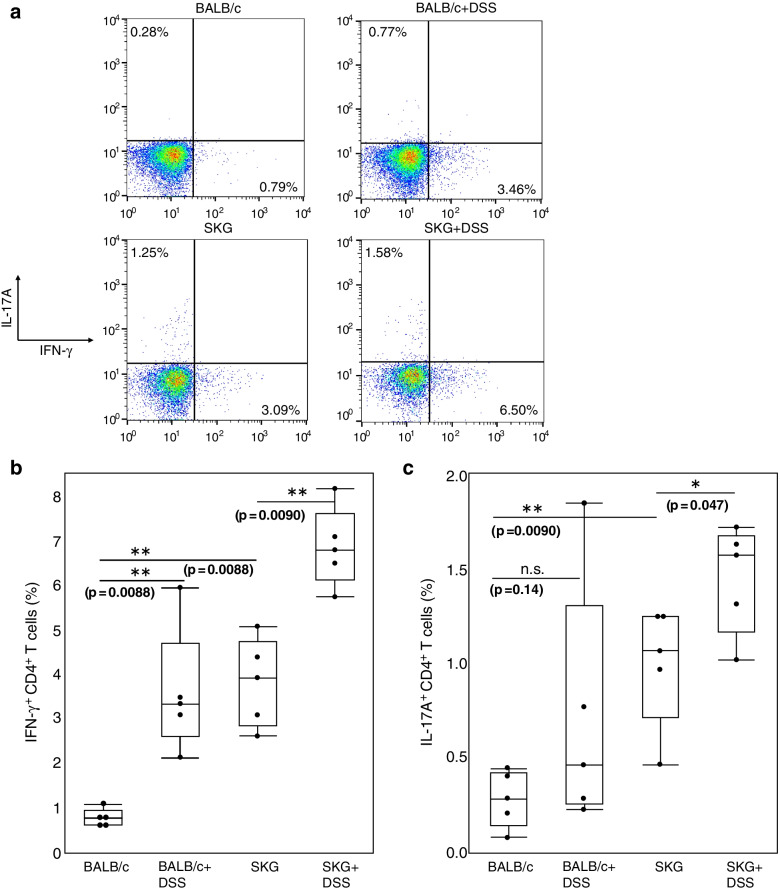
Oral DSS activates Th1 and Th17 immunity in the spleens of SKG mice. **a** Representative flow cytometry (FCM) plots of splenic CD4^+^T cells at weeks 0 and 12. **b**, **c** IFN-γ (**b**) and IL-17A (**c**) positivity of splenic CD4^+^ T cells in BALB/c and SKG mice at weeks 0 and 12. Statistical analyses were performed by using the Mann-Whitney *U* test (*P <0.05, **P <0.01)

We then evaluated the changes in Th cell populations in the spleen, before and after DSS administration in BALB/c and SKG mice (*n*=5 per group). Before DSS administration, SKG mice possessed a significantly higher percentage of both IFN-γ-producing Th cells (Th1 cells) and IL-17A-producing Th cells (Th17 cells) than BALB/c mice. After 2 weeks of DSS administration, the percentage of Th1 cells increased significantly in both BALB/c and SKG mice (*P* <0.01), and that of Th17 cells was significantly increased in SKG mice (*P* <0.05) (Fig. 2a–c). These results suggest that oral DSS administration activates both systemic Th1 and Th17 immunity in SKG mice.

### Antibiotics, not an anti-fungal agent, ameliorate DSS-induced peripheral SpA of SKG mice

**Fig. 3:**
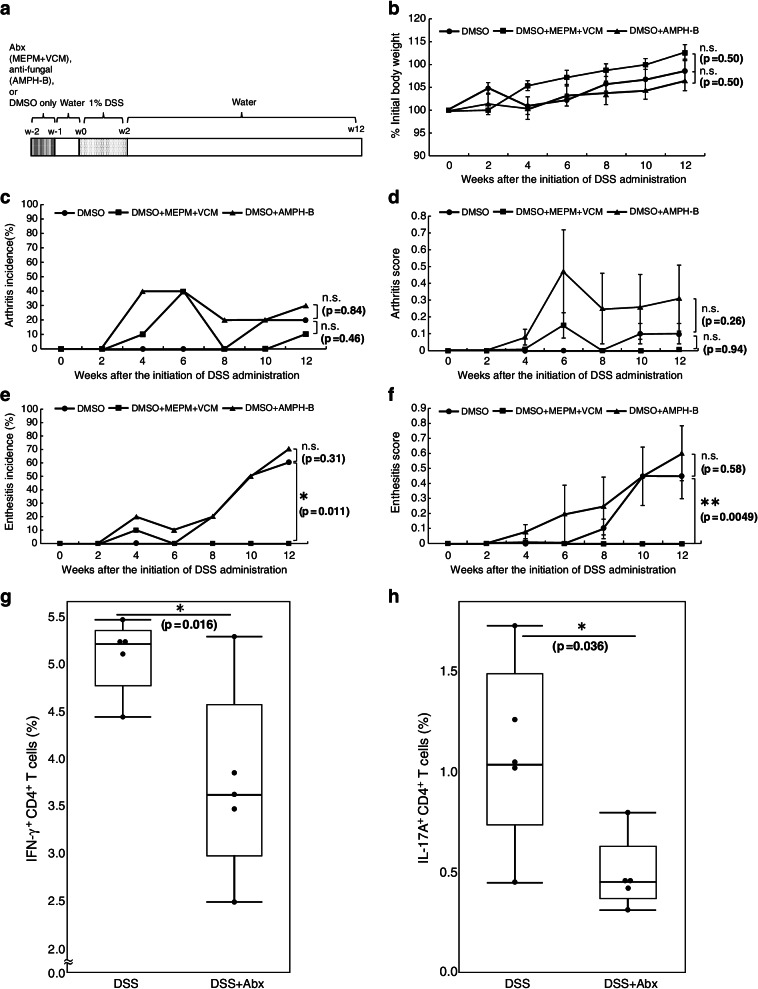
The antibiotics, MEPM and VCM, but not the anti-fungal agent AMPH-B, ameliorate DSS-induced peripheral SpA of SKG mice. **a** Experimental scheme of mouse treatment with oral antibiotics (MEPM (1 g/L)+VCM (0.5 g/L)) or the anti-fungal agent (AMPH-B (0.3 g/L)) and DSS. Agents were administered in their drinking water with 6% DMSO for 1 week, followed by regular water for 1 week. Then, 1% DSS was administered in their drinking water for 2 weeks, followed by regular water. Ten mice were used in each group. **b** The body weight change in each group over the experiment. **c**–**f** Arthritis incidence rates and scores, enthesitis incidence rates and scores were recorded every 2 weeks. Values are the mean ± standard error of the mean (SEM). **g**, **h** FCM analysis of IFN-γ (**g**) and IL-17A (**h**) positivity of splenic CD4^+^ T cells in SKG mice administered with DMSO and DMSO plus antibiotics (MEPM+VCM). Five mice per group were used. Statistical analyses were performed by using the Mann-Whitney *U* test (*P <0.05, **P <0.01)

To investigate which is pivotal, bacterial or fungal translocation in the pathogenesis of DSS-induced pSpA features in SKG mice, we administered antibiotics (MEPM plus VCM) or an anti-fungal agent (AMPH-B) in drinking water for 1 week from the time point 2 weeks before the initiation of DSS administration in another set of cohorts. Six % DMSO was added to the drinking water in the 1st week in each group to dissolve AMPH-B. Female SKG mice aged of 8–12 weeks (*n*=10 per group) were administered DMSO only, DMSO and antibiotics (MEPM and VCM), and DMSO and AMPH-B. After 1 week of free water drinking, 1% DSS in drinking water was administered for 2 weeks. All experiments were conducted under SPF conditions. Body weights, arthritis, and enthesitis scores were recorded every 2 weeks from the initiation of DSS administration over 12 weeks (Fig. 3a). At week 12, there was no significant difference in the rate of change in body weight between SKG mice treated with DMSO only, DMSO plus MEPM and VCM, and DMSO plus AMPH-B (Fig. 3b). There was no significant difference in arthritis incidence rate or scores between the three groups, which could be explained by the anti-inflammatory effect of DMSO (Fig. 3c, d). The incidence rate of enthesitis and enthesitis scores at week 12 was lower (zero) in the group with MEPM and VCM, not with AMPH-B, compared to the control group (Fig. 3e, f). We also examined the difference in the percentage of Th1 and Th17 cell populations among CD4^+^ T cells in the spleen, between the group with MEPM and VCM and the control group after 2 weeks of DSS administration. As a result, the percentage of both Th1 and Th17 cells among CD4^+^ T cells in the spleen was significantly lower in the group with MEPM and VCM than in the control group with DMSO only (Fig. 3g, h). These data indicate that bacterial, not fungal, translocation plays an essential role in the pathogenesis of DSS-induced pSpA in SKG mice.

### Oral DSS increases the bacterial DNA load and the number of bacterial species in whole blood

**Fig. 4:**
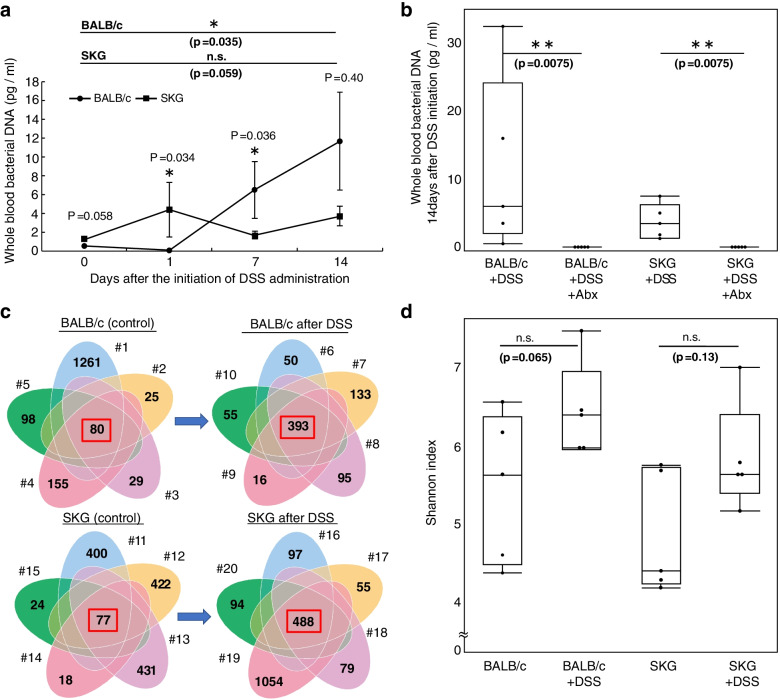
Oral DSS administration increases the amount of circulating bacterial DNA and the number of common species in the groups. **a** The concentration of bacterial DNA in whole blood measured by absolute qPCR, 0, 1, 7, and 14 days after oral DSS treatment initiation. Five mice per group were used. Values are presented as mean ± SEM. **b** The concentration of bacterial DNA measured by qPCR in whole blood after DSS treatment with and without antibiotics. Five mice per group were used. **c** Venn diagrams showing the number of bacterial species in circulating bacterial DNA on days 0 and 14. Five mice were examined per group. Identical numbers of mice are shown by #. Twenty mice were examined as a whole. **d**. The Shannon index before and after DSS administration. Five mice were examined per group. Statistical analyses were performed using the Mann-Whitney *U* test (*P <0.05, **P <0.01)

DNA from whole blood was extracted in another set of cohorts to confirm that bacterial translocation was caused by DSS administration and to investigate its chronological changes. The whole blood of BALB/c and SKG mice before DSS and 1, 7, and 14 days after the initiation of DSS administration were collected (*n*=5 per group). The concentration of bacterial DNA was evaluated by absolute qPCR on the 16S rRNA V3-V4 coding gene. In addition, NGS-based 16S gene analyses were performed to determine the profile of bacterial DNA in whole blood. By qPCR, it was clarified that SKG mice intrinsically have more bacterial DNA in whole blood than BALB/c mice (*p*=0.058), and the discrepancy became even larger on day 1 (*P*=0.034). The concentration of circulating bacterial DNA in BALB/c mice elevated on day 7 with a delay compared to SKG mice (Fig. 4a, Table 1). It was also confirmed that antibiotics (MEPM and VCM) decrease the bacterial DNA load in whole blood (Fig. 4b). The number of bacterial species commonly detected in individual five mice greatly increased after 2 weeks of DSS administration in both BALB/c and SKG mice (80 to 393, and 77 to 458, respectively), with an increase in the Shannon index (not at significant levels) (Fig. 4c, d).

**Table 1:** Ct values of whole blood bacterial qPCR (*n*=5 per group)

	Day 0	Day 1	Day 7	Day 14	Day 14+Abx
BALB/c Mean±SEM	29.7±0.67	31.1±0.85	27.4±0.36	26.8±0.67	33.1±0.58
SKG Mean±SEM	28.1±0.22	28±0.98	28.3±0.45	28.1±0.4	34±0.36
BALB/c Median, IQR	28.9 (28.9-30.6)	30.5 (30.5-30.8)	26.8 (26.8-28.3)	26.8 (26.2-27.0)	33.1 (33.0-33.4)
SKG Median, IQR	28.4 (27.8-28.4)	27.9 (27.4-28.5)	28.6 (27.7-28.7)	28.2 (27.6-28.6)	34 (33.1-35.0)

### Specific bacterial DNA are detected in the whole blood of DSS-treated SKG mice

**Fig. 5:**
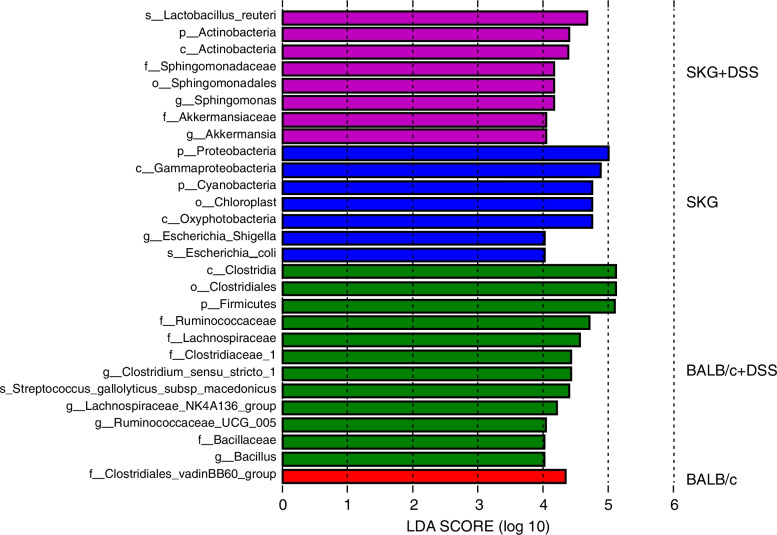
LEfSe analysis of circulating bacterial DNA in the whole blood of BALB/c and SKG mice with and without oral DSS administration. The cutoff was set as LDA score > 4.0

To determine whether bacterial DNA specific to the DSS-treated SKG mouse group was detected in whole blood, an Anosim test and a LEfSe analysis were performed using the same NGS dataset. An Anosim test was performed to examine whether there was a significant beta-diversity between the group of SKG mice with DSS and the other three groups (*n*=5 per group). It clarified that there was a significant beta-diversity between the group of SKG mice with DSS and the group of BALB/c mice without DSS (*r*= 0.6 and *p*= 0.016), the group of BALB/c mice with DSS (*r*= 0.54 and *p*= 0.01), and the group of SKG mice without DSS (*r*= 0.82 and *p*= 0.01) (Table 2). The LEfSe analysis was performed with the same NGS dataset to clarify the bacterial genera and species specific to the group of SKG mice with DSS, setting the significant *p*-value as <0.05, and the cut off of linear discriminant analysis (LDA) score as 4.0 (log 10). As a result, *Lactobacillus reuteri*, *Sphingomonas*, and *Akkermansia* were detected as significantly specific genera or species in the whole blood of the group of SKG mice with DSS (Fig. 5).

**Table 2:** Results of Anosym test (*n*=5 per group)

	R value	***P*** value
**BALB/c vs BALB/c+DSS**	**0.092**	**0.231**
**BALB/c vs SKG**	**- 0.06**	**0.71**
**BALB/c vs SKG+DSS**	**0.6**	**0.016**
**BALB/c+DSS vs SKG**	**0.456**	**0.028**
**BALB/c+DSS vs SKG+DSS**	**0.54**	**0.01**
**SKG vs SKG+DSS**	**0.82**	**0.01**

## Discussion

This study demonstrated that oral DSS administration in SKG mice under SPF conditions solely elicited pSpA features and that this was associated with bacterial, not fungal translocation. First, oral DSS administration solely provokes pSpA features in SKG mice, with bacterial dissemination into the blood. Enthesitis was confirmed in at least 50% of the DSS-administered SKG mice. In addition to peripheral enthesitis, in DSS-treated SKG mice, axial lesions were also confirmed adjacent to the vertebral disk at pathological level, although the cell infiltration level was much milder than that reported in curdlan-treated SKG mice [10]. This oral DSS-induced pSpA features in SKG mice were suppressed by antibiotics (MEPM plus VCM), which are known as broad-spectrum antibiotics, not by AMPH-B, which is also known as a broad-spectrum anti-fungal agent. These results suggest that bacterial factors are more important than fungal factors in the pathogenesis of oral DSS-induced pSpA. In fact, several reports have shown that antibiotics might be effective in reducing the disease severity of SpA [14, 15]. Other reports have shown that antibiotics also have therapeutic potential for the treatment of IBDs [16, 17].

Arthritis scores unexpectedly improved even in the control group with DMSO only, which might be explained by that DMSO is known for its therapeutic effects on arthritis and other inflammatory diseases [18–21]. DMSO did not influence the incidence rate of enthesitis and its score in the control group. This suggests that there are different mechanisms between DSS-induced arthritis and enthesitis. Arthritis, in a narrow sense, means intraarticular inflammation such as synovitis which is well-known for the pathology of rheumatoid arthritis. Comparing to arthritis in a narrow sense, enthesitis has been known for its closer relationships with local mechanical stress and tissue resident immune cells which utilise pattern recognition receptors at extra-articular sites [22]. Bacterial components including CpG-rich DNA might stimulate immune cells [23], and the same phenomena can happen in our DSS-treated SKG mouse model, but further research is required.

We also investigated changes in Th1 and Th17 cell levels in the spleen, because they are implicated in the systemic type 1 and type3 immunity, respectively. It has been reported that intraperitoneal injection of β-glucan increases Th17 cells 3–4-folds in the lymph nodes of SKG mice, and that T cells sorted from normal SKG mice possess the potential to cause arthritis in recipient RAG2 KO mice, but not from IL-17A KO SKG mice. Therefore, Th17 cells play pivotal roles in the pathogenesis of arthritis in SKG mice [8]. In this study, it is important to note that the percentage of Th1 cells increased by the same or more magnitude as Th17 cells (the increase ratios calculated using the medians were 1.73 and 1.48 folds, respectively), and the observed increase in Th1 cells was more significant than that in Th17 cells in our DSS-treated SKG mice (*p*= 0.009, 0.047, respectively). Increases in Th1 cell percentages have also been observed in wild-type mice treated with DSS [24, 25]. These findings suggest that SKG mice, which have a T cell signal dysfunction, can manifest different phenotypes depending on the environment and adjuvant. In fact, many of autoimmune/autoinflammatory diseases and immune deficiency diseases in human have been known to be associated with T cell signal dysfunction [26, 27]. Our studies support that enteritis stimulates both systemic Th1 and Th17 immunity via bacterial translocation in the gut, and elicits SpA pathogenesis in those with mutations associated with T cell signal transduction.

We also demonstrated that SKG mice had more bacterial DNA in whole blood than BALB/c mice. Especially, 1 day after administering oral DSS administration, SKG mice contained more bacterial DNA in whole blood than BALB/c mice. These findings might support a role for T cell signal dysfunction in the gut vulnerability, or “leaky gut” of SKG mice. However, there was no significant difference in the amount of bacterial DNA in whole blood between BALB/c and SKG mice on day 14. Therefore, there must be “the second hit” in the SpA pathogenesis of DSS-treated SKG mice in addition to the gut vulnerability. Then, we examined the diversity of circulating bacterial DNA. The number of bacterial species which were commonly detected in all mice per group were almost the same 14 days after DSS administration between BALB/c and SKG mice, and the alfa-diversity shown by the Shannon index even increased in both groups (not at significant levels). Therefore, beta-diversity could be the clue, and whether there are bacterial genera and/or species specific to the whole blood of the group of SKG mice treated with DSS was of our great interest.

Then, we performed an Anosim test and a LEfSe analysis. An Anosim test, which indicates beta-diversity, revealed that the group of SKG mice with DSS had significantly unique bacterial species’ DNA in their whole blood, compared to any other three groups: BALB/c mice before DSS administration, BALB/c mice after DSS administration, and SKG mice before DSS administration. Moreover, using a LEfSe analysis, DNAs of *Lactobacillus reuteri*, *Sphingomonas*, and *Akkermansia* were detected as significantly increased bacteria with a high LDA score (LDA > 4.0 (log10)) in the whole blood of SKG mice with DSS administration. *Lactobacillus reuteri* is implicated as a type I interferon (IFN) inducer and can exacerbate autoimmune colitis and autoimmune diseases, such as systemic lupus erythematosus (SLE) [28, 29]. *Sphingomonas* produces sphingolipids instead of lipopolysaccharide (LPS) and can stimulate NKT cells [30, 31]. *Akkermansia* feeds on mucin, which is known for its anti-inflammatory effects, but some studies have shown they can exacerbate inflammation and contribute to autoimmune diseases [32–34]. These bacteria might be key players in the pathogenesis and promising therapeutic targets in SpA treatment, and further research is required.

Our study has some limitations. First, all the experiments were conducted in a single facility. It is known that commensal microbial diversity depends on each animal facility. Second, we have not completely excluded non-germicidal effects of antibiotics, although we did not use antibiotics which have strong anti-inflammatory effects such as macrolides and tetracyclines, rifampin, and metronidazole [35, 36]. Moreover, this study was limited to female mice. (We have observed similar phenotypes in male mice, but data were not shown.)

## Conclusions

We have demonstrated that oral DSS administration solely elicits pSpA features in SKG mice with specific bacterial translocation, and systemic Th1 and Th17 activation. This was suppressed by antibiotics, not by the anti-fungal agent. This study adds to the growing body of evidence that gut bacterial translocation plays a pivotal role in the pathogenesis of SpA.

## Data Availability

The datasets used and/or analysed during the current study are available from the corresponding author on reasonable request.

## Abbreviations

Abx: Antibiotics
AMPH-B: Amphotericin B
Anosim: Analysis of similarities
AS: Ankylosing spondylitis
DMSO: Dimethyl sulfoxide
DSS: Dextran sulfate sodium
FCM: Flow cytometry
H&E: Haematoxylin and eosin
IBD: Inflammatory bowel disease
IFN: Interferon
LDA: Linear discriminant analysis
LEfSe: Linear discriminant analysis effect size
LPS: Lipopolysaccharide
MEPM: Meropenem trihydrate
MSK: Musculoskeletal
NGS: Next-generation sequencing
PsA: Psoriatic arthritis
pSpA: Peripheral spondyloarthritis
qPCR: Quantitative polymerase chain reaction
ReA: Reactive arthritis
SLE: Systemic lupus erythematosus
SpA: Spondyloarthritis
SPF: Specific pathogen free
VCM: Vancomycin hydrochloride
